# Implementing a pilot leadership course for internal medicine residents: design considerations, participant impressions, and lessons learned

**DOI:** 10.1186/s12909-014-0257-2

**Published:** 2014-11-30

**Authors:** Daniel M Blumenthal, Ken Bernard, Traci N Fraser, Jordan Bohnen, Jessica Zeidman, Valerie E Stone

**Affiliations:** Department of Medicine, Massachusetts General Hospital, Harvard Medical School, 55 Fruit Street, GRB 800, Boston, MA 02114 USA; Division of Cardiology, Department of Internal Medicine, Massachusetts General Hospital, Harvard Medical School, Boston, MA USA; Department of Emergency Medicine, Partners Healthcare, Harvard Medical School, Boston, MA USA; Division of General Internal Medicine, Massachusetts General Hospital, Harvard Medical School, Boston, MA USA; Department of General Surgery, Massachusetts General Hospital, Harvard Medical School, Boston, MA USA; Department of Medicine, Mount Auburn Hospital, Harvard Medical School, Boston, MA USA

**Keywords:** Leadership development, Management, Quality of care, Teamwork, Patient safety

## Abstract

**Background:**

Effective clinical leadership is associated with better patient care. We implemented and evaluated a pilot clinical leadership course for second year internal medicine residents at a large United States Academic Medical Center that is part of a multi-hospital health system.

**Methods:**

The course met weekly for two to three hours during July, 2013. Sessions included large group discussions and small group reflection meetings. Topics included leadership styles, emotional intelligence, and leading clinical teams. Course materials were designed internally and featured “business school style” case studies about everyday clinical medicine which explore how leadership skills impact care delivery. Participants evaluated the course’s impact and quality using a post-course survey. Questions were structured in five point likert scale and free text format. Likert scale responses were converted to a 1-5 scale (1 = strongly disagree; 3 = neither agree nor disagree; 5 = strongly agree), and means were compared to the value 3 using one-way T-tests. Responses to free text questions were analyzed using the constant comparative method.

**Results:**

All sixteen pilot course participants completed the survey. Participants overwhelmingly agreed that the course provided content and skills relevant to their clinical responsibilities and leadership roles. Most participants also acknowledged that taking the course improved their understanding of their strengths and weaknesses as leaders, different leadership styles, and how to manage interpersonal conflict on clinical teams. 88% also reported that the course increased their interest in pursuing additional leadership training.

**Conclusions:**

A clinical leadership course for internal medicine residents designed by colleagues, and utilizing case studies about clinical medicine, resulted in significant self-reported improvements in clinical leadership competencies.

**Electronic supplementary material:**

The online version of this article (doi:10.1186/s12909-014-0257-2) contains supplementary material, which is available to authorized users.

## Background

Mounting evidence indicates that clinical leadership—defined as leadership by practicing clinicians at the point of care—is an important determinant of health care quality and cost control [1]. However, clinical leadership skills, including team leadership abilities, relationship management, emotional intelligence, situational leadership, and the capacity for reflection, are not systematically emphasized in medical school or post-graduate training in the United States (U.S.) [1-13]. Consequently, many clinicians trained in the U.S. develop these competencies through ad-hoc, on-the-job learning [14,15]. Interest in providing physicians with formal leadership training has grown as physicians, educators, researchers, and policy makers have started to recognize the importance of these skills [1].

The transition from intern year to the second, or junior, year of internal medicine (IM) residency is accompanied by significant changes in residents’ clinical and leadership responsibilities. Junior residents take on many critical clinical leadership roles, including leading multidisciplinary clinical teams on general medical services and in the intensive care unit (ICU). Several U.S. IM residency programs—including the Cleveland Clinic and the University of Washington—provide focused leadership training to help support residents through this transition. These interventions are associated with meaningful improvements in participants’ leadership skills and confidence in assuming clinical leadership roles [4,11]. Prior to the 2013-14 academic year, the Massachusetts General Hospital’s (MGH) Department of Medicine (DOM) offered residents some formal opportunities to build leadership skills, but did not provide them with systematic, classroom-based clinical leadership training.

In 2013, the MGH designed and implemented a pilot Leadership Development Course (LDC) for second year IM residents. The LDC’s goals were to: 1) Help residents to develop basic leadership skills that are directly applicable to their clinical work; 2) Promote residents’ personal and professional development; and 3) Build longer-term interest in leadership and management. The aims of this paper are three-fold: First, we describe the methods used to develop, implement, and evaluate this pilot leadership course; second, we present initial post-course feedback from participants; and third, we highlight lessons from our experience that may inform efforts to create similar training interventions in other residency programs and across specialties.

## Methods

### Setting

The MGH is a 1057 bed teaching hospital which is affiliated with Harvard Medical School and is part of Partners Healthcare, a not-for-profit multi-hospital health care system that owns ten Massachusetts hospitals. The MGH DOM residency program includes approximately 65 interns, 55 junior residents, and 55 third year residents. Each intern class includes approximately ten “preliminary” interns who are completing one year of IM training prior to their dermatology, neurology, psychiatry, radiology, ophthalmology, or anesthesiology residency. The MGH DOM residency training program emphasizes four major development goals: Clinical excellence, teamwork skills, leadership development, and career development [16].

### Designing the leadership development course

#### Course origins and participants in course design

Three of this paper’s authors (DB, KB, and JB) undertook a year-long field study of leadership development in health care while students at Harvard Business School (HBS). This project, which was advised by an HBS Professor and the Medical Director of the MGH Physician’s Organization (MGPO), produced a preliminary outline of the LDC. The outline was subsequently refined in consultation with DOM faculty. Eleven IM residents were recruited to help develop course content.

#### Guiding principles and frameworks

We used two frameworks to help guide the LDC’s development. First, we adhered closely to a set of nine established best practices for designing effective leadership training interventions (Table 1) [1,17-20]. Second, the course employs an iterative, three part process of experience, reflection, and feedback which has proven effective in other management education interventions [19].

**Table 1:** Elements of LDC that reflect “Best Practices” of effective leadership development courses*

**Design principle**	**LDC course elements that reflect principle**
**Reinforce a supportive culture**	• Involve residents and key faculty stakeholders in course’s development.
	• Course timed to support significant transition for residents.
	• All course discussions are strictly confidential.
**Ensure high-level sponsorship and involvement**	• Early, conscious effort to cultivate support from key departmental and hospital leaders.
	• Key stakeholders involved in course design, received routine progress reports.
	• Course received seed funding from department.
	• Faculty, including Chief of DOM, taught course.
**Tailor the program’s goals and approach to its context**	• Course’s goal: Help residents to build clinical leadership skills necessary to excel in upcoming supervisory roles.
	• Case study method is interactive, and simulates real life decision-making.
	• Course taught during “lighter,” outpatient rotation; limited outside preparation.
	• Case discussions led by internal clinicians-leaders familiar with work environment and residents’ development needs.
**Target the program towards specific groups**	• Targeted towards Internal Medicine (IM) residents during transition from intern to junior year.
	• Resident input into curriculum development helped to ensure relevant, practical, and engaging content.
**Integrate all features of the program**	• Course material (case studies, large group meetings, small group exercises, supplementary reading material) organized by discrete sessions focusing on individual leadership styles and building to leadership within teams.
	• Each course session included, and reinforced, iterative process of experience, reflection, and feedback.
**Offer extended learning periods with support**	• Faculty was available for follow up discussions after course’s conclusion.
	• Course’s developers offered to provide residents with additional learning materials at their request.
**Employ multiple learning and teaching methods**	• Course participants learned through reading relevant literature, reading and discussing case studies, self-reflection, didactic teaching, and role plays.
**Encourage ownership of self-development**	• Participants informed during first session that course’s success, and their individual and group learning, was dependent on participation and engagement.
**A commitment to continuous improvement**	• Multimodal course evaluation strategy that is assessing many different outcomes.
	• Post-course survey data being used to revise course syllabus.
	• Needs assessments to be administered to all interns each year to gather information about specific leadership development needs.

*Sources: Blumenthal et al. [1] and McGonagill and Pruyn [17].

#### Central considerations related to course design and implementation

We identified a number of important questions and considerations regarding the course’s design and implementation, which are described below:

- Course Length, Timing, and Size
  The course’s timing and length were designed to overcome logistical challenges typical of administering educational curricula during residency. It was important to find a recurring time block when participating residents did not have competing clinical commitments. Thus, we realized that, due to the work load and scheduling inflexibility of inpatient rotations, the course would have to be scheduled during outpatient or elective time. We therefore had two viable scheduling options: 1) Incorporating the curriculum into one of three yearly ambulatory care rotations (ACR), or 2) creating a two-week course which residents could take during an elective block.
  We also considered during which period of residency training residents would derive the most benefit from leadership training. For example, offering the course early in residents’ junior years would deliver this training just as residents were preparing for new clinical leadership roles, which could improve the course’s efficacy and also increase resident engagement in the course. Furthermore we considered whether the pilot should be offered to all residents, or a portion of them. Given the impossibility of simultaneously excusing all residents from clinical responsibilities, reaching all residents would pose large logistical and scheduling challenges. Moreover, including all residents in the course would prevent us from evaluating course outcomes using a case-control study design in which residents who didn’t take the course were compared to course participants. In addition, we deliberated over whether the course be required or optional. While making the course required would ensure adequate participation, some residents might resent being forced into the course. Alternatively, residents could be automatically enrolled and given the opportunity to opt out if they had a legitimate reason to do so (e.g. previous extensive leadership training in business school or the military).
- Curriculum Design
  We confronted an additional decision and set of tradeoffs as we started to design the curriculum: Should we purchase access to externally developed course materials, build the course ourselves, or take a hybrid approach? Using an external course afforded several benefits. Indeed, most external courses were developed by leadership experts and vetted by prior users, and purchasing materials would save time. However, external course materials were also expensive. Moreover, we encountered few external courses which focused on clinical leadership challenges that residents commonly faced at this point in their training. Building the course internally would enable us to tailor it towards trainees’ particular leadership development needs, and to show them how leadership skills would directly impact their work—which have been shown to be important determinants of the success of leadership training [1]. Furthermore, involving residents in the course’s design might help to build support for the course among residents.
- Choosing Educators
  While identifying potential teachers for the LDC, we considered a few questions, including: 1) Should we recruit facilitators from within the MGH (“internal”), from outside the MGH (“external”), or both?; and 2) Should all session leaders be practicing clinicians? While external leadership experts might have more experience with teaching leadership skills, internal facilitators, and particularly practicing clinicians, would better understand the specific leadership challenges that residents face, and residents’ relative leadership deficits.

#### Final course timing, participants, and length

The inaugural version of the LDC was offered to seventeen new junior residents during a month-long ACR rotation in July 2013—their first month as juniors. While all primary care residents in a class are scheduled for the same ACR rotations, all other DOM residents are randomly assigned to ACR rotations. Thus, all primary care junior residents took the LDC, whereas a random subset of all other junior residents were enrolled. The course was offered only once during the 2013-14 academic year. The course syllabus did not include formal plans for post-course education, but additional reading was made available to participants at their request.

#### Final course structure, content, and educators

The course consisted of a weekly two to three hour session for four weeks. Course sessions addressed the following four topics: 1) Introduction to Clinical Leadership and Leadership Styles (sessions one and two); 2) Authentic Leadership (session two); 3) Leading with Emotional Intelligence (session three); and 4) Leading Clinical Teams (session four) (Additional file 1: Table S1). The LDC included both large group discussions and small group meetings. Large group discussions focused on case studies, videos about physician leadership (session 3 only), and role plays (session 3 only). Large group discussions in sessions one and four were facilitated by DOM faculty—including the Chief of the DOM—while session three was led by a Professor of Psychiatry and chief resident in Psychiatry with prior experience teaching about emotional intelligence. We intentionally chose facilitators who had a diverse array of prior and current leadership experiences. All facilitators were practicing physicians, highly respected as clinicians and teachers, and had extensive experience working with residents and leading clinical teams.

Participants were pre-assigned to groups of four to five residents which met weekly during the course (without faculty). Participants were not allowed to switch into groups other than their assigned group. Small group meetings were designed to facilitate more intimate and protected discussion about course concepts and reflection on their application to clinical practice. Small group discussions were strictly confidential.

The LDC’s syllabus and content were developed by residents, with help and support from DOM faculty and a chief resident. Case studies, which were generally three to five pages long and focused on everyday clinical scenarios, were structured like business school case studies and highlighted how core leadership concepts were relevant to daily clinical practice. Core concepts and frameworks addressed in these cases included: Clinical leadership [1], Goleman’s six leadership styles [21] and five components of emotional intelligence [22], authentic leadership (as defined by George) [19], and Hackman’s model of effective team leadership [23]. Residents prepared teaching notes for each case which included background information about relevant case concepts, theoretical frameworks, and teaching points. Teaching notes were reviewed with faculty facilitators prior to their course sessions. Required preparation for each session was expected to take 30 minutes or less to complete. Residents completed short exercises to prepare for two different small group meetings.

### Course evaluations

#### Development of evaluation framework

We used a model of Kirkpatrick’s four levels of evaluations for educational interventions adapted for health care interventions to guide the design of our evaluation framework [24]. We conducted literature searches in PubMed, Proquest, and online to identify published studies of assessments of leadership training needs, leadership skills, and evaluations of leadership training interventions. These searches identified a small number of studies of leadership training interventions for clinicians [3-8,11,12,25-28]. Few studies attempted to evaluate clinical or financial outcomes, or included rigorously validated survey instruments [11,29-32]. Multiple validated instruments for evaluating leadership quality were identified in studies conducted outside of health care, none of which were validated in physician cohorts [33-35]. Before the course began, we finalized a list of outcomes to evaluate, which included: Participants’ reactions to the LDC (Kirkpatrick Level 1) and participants’ knowledge about and attitudes towards leadership skills and leadership development (Kirkpatrick Levels 2a-b).

#### The post-course survey

We assessed participants’ immediate reactions to the course with a survey which was administered to all LDC participants after the course’s final session (Table 2). This survey included questions about the course’s relevance to participants’ clinical roles and responsibilities, the effectiveness of different teaching methods and facilitators, and whether participants felt more prepared to address clinical leadership challenges after taking the LDC. Responses to these questions were structured in five point likert scale format (“Strongly Agree,” “Somewhat Agree,” “Neither Agree Nor Disagree,” “Somewhat Disagree,” and “Strongly Disagree”). The survey also included free text questions which asked respondents about the course’s strengths, weaknesses, and suggestions for improvement. An additional free text question asked participants why they would or wouldn’t recommend the course to colleagues. The survey was designed by two residents and edited by Eric Campbell, Professor of Medicine at Harvard Medical School and a survey design expert. A third year IM resident reviewed the survey, and deemed it to have adequate clarity, face validity, and content validity. Surveys were administered through RED Cap, a secure online data repository (REDCap, Vanderbilt University, Nashville, TN). All survey responses were de-identified; participants were told that completion of a survey implied informed consent to use this information for research purposes. This study was evaluated and deemed exempt by the MGH’s Institutional Review Board (IRB).

**Table 2:** **Course participants’ evaluations of leadership development course**

**Survey question**	**Mean Likert scale response**	P-value*	**Percent who strongly or somewhat agree with statement**
Overall, the leadership course provided content that is relevant to my practice of clinical medicine.	4.88	<0.0001	100%
Overall, the leadership course provided skills that are relevant to my practice of clinical medicine.	4.81	<0.0001	100%
Session I (Introduction & Core Leadership Styles) provided content that is relevant to my practice of clinical medicine.	4.88	<0.0001	100%
Session I (Introduction & Core Leadership Styles) provided skills that are relevant to my practice of clinical medicine.	4.69	<0.0001	100%
Session III (Leading with Emotional Intelligence) provided content that is relevant to my practice of clinical medicine.	4.56	<0.0001	88%
Session III (Leading with Emotional Intelligence) provided skills that are relevant to my practice of clinical medicine.	4.44	<0.0001	88%
Session IV (Leading Clinical Teams) provided content that is relevant to my practice of clinical medicine.	4.88	<0.0001	100%
Session IV (Leading Clinical Teams) provided skills that are relevant to my practice of clinical medicine.	4.75	<0.0001	100%
The evening small group session about leadership styles provided content that is relevant to my practice of clinical medicine.	4.25	<0.0001	94%
The evening small group session about leadership styles provided skills that are relevant to my practice of clinical medicine.	4.25	<0.0001	88%
The large group case discussions were an effective way to present course topics.	4.81	<0.0001	100%
The small group meetings contributed significantly to my learning during this course.	4.13	0.0009	75%
As a direct result of the DOM leadership course, I have a better understanding of my own strengths and weaknesses as a leader.	4.63	<0.0001	94%
As a direct result of the DOM leadership course, I have a better understanding of different leadership styles.	4.69	<0.0001	94%
My knowledge of core leadership styles will inform my interactions with my clinical teammates.	4.63	<0.0001	100%
As a direct result of the DOM leadership course, I feel more prepared to face challenges that arise with team members below me (e.g. residents, medical students, etc…).	4.69	<0.0001	100%
As a direct result of the DOM leadership course, I feel more prepared to face challenges that arise with team members at my level (e.g. my co-residents).	4.50	<0.0001	94%
As a direct result of the DOM leadership course, I feel more prepared to face challenges that arise with team members above me (e.g. fellows, attendings, etc…).	4.44	<0.0001	94%
As a direct result of the DOM leadership course, I feel more prepared to face challenges that arise with non-physician colleagues (e.g. nurses, case managers, physical therapists, nutritionists, etc…).	4.44	<0.0001	88%
Taking this leadership course has increased my interest in pursuing additional leadership training and development opportunities.	4.38	<0.0001	88%
I plan to pursue additional leadership training and development opportunities in the future.	4.06	0.002	75%
I would recommend this course to my colleagues.	4.81	<0.0001	94%
All junior residents should be required to take this course.	4.38	0.0002	81%

*P-values are one sided, and compare the means of participants’ likert scale responses to the number 3, which corresponds to the likert scale answer “neither agree nor disagree.”

#### Data analysis

Participants’ responses to likert scale questions were assigned a numerical value ranging from one (“Strongly Disagree”) to five (“Strongly Agree”). Using Microsoft Excel 2013 (Microsoft Corp., Redmond, WA), we calculated mean response values for each question, and performed one-way T tests to determine if the mean value of all responses to a question was significantly different from three—the value corresponding to “Neither Agree Nor Disagree.”

Free text responses to questions about course strengths and improvement needs were analyzed using the comparative source method [36,37]. Two co-authors independently identified themes in participants’ responses, then iteratively compared and refined their theme lists until they agreed on a common set of themes (Additional file 1: Tables S2-S3). Next, the evaluators independently quantified the number of distinct references to each theme in the responses. They repeatedly compared and revised their reference lists until they agreed on the number of references to each theme.

## Results

### Course participants

Sixteen residents took the course (one resident, who had extensive prior leadership training and experience, opted out). Seven residents were in the primary care residency program (one of whom was in the global primary care residency program), eight were in the categorical IM program, and one resident was in the combined medicine-pediatrics residency program (Table 3).

**Table 3:** **Characteristics of leadership course participants**

**Characteristic**	**N (%)**
Age	
26-30	11 (68.7)
31-35	5 (31.3)
Gender	
Male	10 (62.5)
Female	6 (37.5)
Ethnicity	
Caucasian	9 (56.3)
Black	1 (6.3)
Asian	6 (37.5)
Marital Status	
Single	5 (31.3)
Married	11 (68.7)
Residency training program	
Categorical	8 (50)
Primary care	6 (37.5)
Global primary care	1 (6.3)
Medicine/Pediatrics	1 (6.3)
Non-MD advanced degree	
PhD	2 (12.5)
MPH	3 (18.8)
MPP	1 (6.3)
Career plan	
General internal medicine	8 (50)
Subspecialty	8 (50)

MPH = Masters in public health; MPP = Masters in Public Policy.

All sixteen LDC participants completed the post-course survey (100% response). The vast majority of participants strongly or somewhat agreed that the LDC provided both content and skills that were relevant to their practice of clinical medicine (Table 2). Participants overwhelmingly felt that large group case discussions were an effective way to present course material, and many indicated that small group meetings also contributed to their learning.

**Figure 1:**
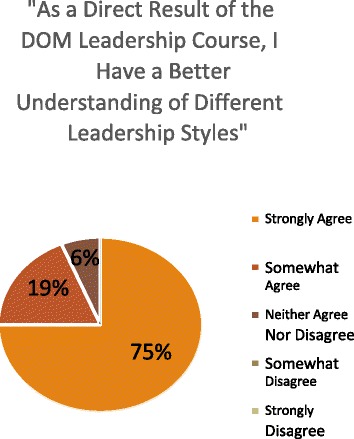
**Impact of leadership course on participants’ understanding of leadership styles.** LDC participants’ likert scale responses to the question “As a direct result of the DOM Leadership course, I have a better understanding of different leadership styles.”

**Figure 2:**
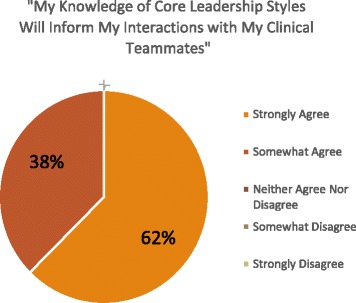
**Participants’ perceptions of relevance of knowledge about different leadership styles.** LDC participants’ likert scale responses to the question “My knowledge of core leadership styles will inform my interactions with my clinical teammates.”

**Figure 3:**
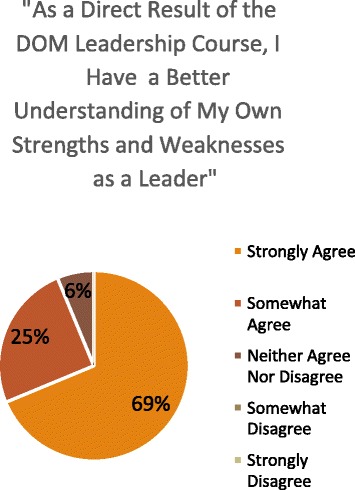
**Impact of leadership course on participants’ awareness of their own strengths and weaknesses as leaders.** LDC Participants’ likert scale responses to the question “As a direct results of the DOM Leadership Course, I have a better understanding of my own strengths and weaknesses as a leader.”

**Figure 4:**
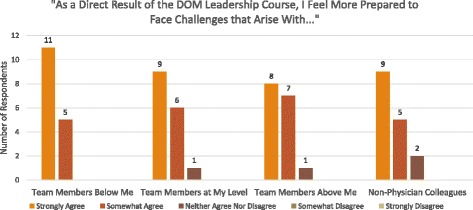
**Impact of leadership course on participants’ perceived ability to manage interpersonal challenges.** LDC participants’ likert scale responses to post course survey questions about their preparedness to face challenges that arise with team members below them, team members at their level, team members above them, and non-physician colleagues.

Most participants also agreed that the LDC helped them to gain a better understanding of their “strengths and weaknesses as a leader” and of “different leadership styles.” Similarly, the majority of participants believed that this improved knowledge of core leadership styles would inform their interactions with clinical teammates (Figures 1, 2, and 3). Moreover, most participants indicated that, as a direct result of the DOM leadership course, they felt more prepared to face challenges arising with team members below them, at their level, and above them, and with non-physician colleagues (Figure 4). Eighty eight percent of participants also strongly or somewhat agreed that taking the LDC increased their interest in pursuing additional leadership training, while 94% indicated that they would recommend the course to colleagues.

### Course strengths

Participants’ comments about course strengths focused on the quality of the large group meetings (ten comments), the quality of reading assignments (five comments), the utility of small group meetings (eight comments), practical skills learned from the course (four comments), and the importance of providing a protected, safe forum for reflection and discussion (four comments) (Additional file 1: Table S2). Residents noted that the course “practically addressed and anticipated issues that we will face as” junior residents, and that the course’s “timing [was] perfect—right before we embark on junior year.” Another resident commented that the course “facilitated my understanding of my authentic leadership style and enabled me to reflect on situations where I am forced out of my authentic style…. I… understand why I feel uncomfortable in these settings and am able to adjust my perspective to improve upon those situations.”

### Course weaknesses and recommendations for improvement

Participants commonly identified the structure and content of the “Leading with Emotional Intelligence” Session as a significant course weakness. One course participant commented, “The emotional intelligence session provided a great platform to discuss the topic, but the lecture, videos, and role playing was quite redundant.” Several residents noted that small group meetings were not as effective as large group discussions, and that small group conversations frequently strayed off topic (Additional file 1: Table S3).

## Discussion

Leadership skills are essential for addressing many challenges faced by health care systems around the world [38-43]. However, many residency training programs, including U.S. IM training programs, have not systematically prioritized leadership training for residents [1,4,11,38,42]. We successfully designed and piloted a multi-session clinical leadership course for IM residents at a large U.S. Academic Medical Center (AMC) that is part of a multi-hospital system. The LDC was designed and implemented internally, taught by respected clinician-leaders, and timed to coincide with residents’ transition from internship to their junior year of residency—a change accompanied by increased clinical leadership responsibilities. Participants reported gains in important leadership skills and knowledge after taking the course—including increased awareness of their strengths and weaknesses as leaders and a better understanding of different leadership styles—and improvements in certain clinical leadership skills, including the ability to address interpersonal challenges [2,24]. According to participants, the LDC’s major strengths included the quality of large group discussions, small group meetings, and reading assignments, a focus on building practical skills, and dedicated time for reflection and confidential discussion.

Participants’ evaluations suggest that the LDC achieved our stated goals of helping trainees to build practical clinical leadership skills; promoting participants’ personal and professional development; and stimulating interest in leadership and management. Participants’ reported gains in skills and knowledge and anticipated changes in leadership behaviors are consistent with prior studies of leadership training interventions for physician faculty (not residents) [2]. The LDC also contained unique design elements, including a presentation about evidence that leadership impacts clinical outcomes and serial meetings with pre-assigned small groups. Perhaps most notably, to our knowledge, ours is the first publication to describe the use of business-school style case studies about everyday clinical practice to teach IM residents about leadership [4,8,9,11,12,44]. The case studies, and discussions about them, were universally viewed as course strengths. Thus, the LDC pilot provides initial “proof of principle” of a new method for teaching leadership to residents. The case study method’s strengths—including its focus on analyzing complex, real-life problems that lack simple solutions; simulating decision-making; and debating alternative viewpoints with colleagues—make it well suited for teaching residents about clinical leadership challenges. Moreover, adult learners, including physicians, often learn best through experiential modalities, including case discussions and simulations [2].

A number of elements were critical to the LDC pilot’s success. First, and consistent with prior work, we found that institutional support for the pilot was essential [2]. In our case, key departmental and institutional leaders backed the pilot from its earliest stages, and provided the course’s designers with autonomy to develop content with limited faculty oversight. Second, we tailored our educational intervention to meet learners’ immediate developmental needs, and to accommodate certain characteristics of the local environment. To maximize participants’ engagement, and the course’s value to them, we delivered the course during residents’ transition from intern year to junior year. At this juncture, participants were nervous about taking on greater leadership responsibilities as junior residents, and were thus eager to improve their leadership skills [4]. We also limited assignments to small amounts of high yield reading to avoid potential conflicts with participants’ clinical responsibilities. Moreover, rather than invite leaders of health systems, corporate executives, or business school professors to teach the pilot course, we recruited teachers from an internal pool of well-respected, experienced clinician-educators and clinician-leaders who were familiar with the leadership challenges that junior residents would likely face.

Third, our decisions to design the course internally, and recruit internal faculty to teach in it, afforded other key benefits. For one, these choices created important opportunities for collaboration between residents designing the course, other residents, and faculty. These opportunities strengthened existing stakeholders’ trust and backing, broadened support for the course, and improved its quality. Moreover, these decisions minimized the course’s financial costs and freed up resources to help finance course evaluation efforts.

The two most commonly cited course weaknesses were the content and lack of structure of small group meetings, and the emotional intelligence session. In our view, participants’ criticisms of the small group sessions may reflect a need to modify the session agendas and provide better guidance to participants about them. Furthermore, we didn’t train course participants to facilitate these meetings or assess participants’ effectiveness as small group leaders, and it is possible that criticisms of the small group sessions reflect ineffective efforts to facilitate them. While we cannot exclude that the small group format itself is ineffective, the large number of positive comments about the small group format suggests that it would be premature to jump to this conclusion. The emotional intelligence session was designed and taught by non-IM faculty members, and included videos and role plays, rather than a case discussion. Most criticisms of this session focused on its structure, exercises, and the facilitators’ teaching styles, rather than the topic(s) being addressed in the session. However, we cannot rule out that residents found learning about emotional intelligence less valuable than other topics presented in the course.

While preliminary and limited in scope, our experience and results have implications for efforts to improve health care quality. Our work suggests that the LDC may help participants to develop important team leadership competencies—including an understanding of different leadership styles, situational leadership, self-awareness, and how to manage interpersonal conflict. Furthermore, we found that the pilot course increased participants’ interest in pursuing future leadership training and opportunities. This result—which is consistent with prior evaluations of leadership development interventions for IM faculty—is important because many healthcare systems desperately need more qualified physician leaders and managers who are interested in assuming formal leadership and management roles [1,8,41,45].

The LDC pilot, and our efforts to evaluate it, have a number of important limitations. We hope that our work can serve as a guide for efforts to create leadership development interventions for physician-trainees at other institutions. However, similar efforts may not be feasible at institutions which lack high level support for leadership training, or staff with expertise in, or a willingness to focus on learning how to teach, leadership development. In addition, we did not provide course participants with formal opportunities for ongoing learning and development [2]. Moreover, we did not evaluate the reliability, internal validity, or external validity of our post-course survey. Furthermore, our other survey instruments are adapted largely from physician surveys that have not been validated among IM residents. Our evaluation strategy also does not include direct measures of health care quality or organizational performance as outcomes. Finally, our course evaluations may be underpowered to identify differences in self-assessed or observed leadership behaviors between course participants and other residents.

To date, few thorough evaluations of paradigms and curricula for teaching leadership to residents, generally, and IM residents in particular, have been undertaken [1,4,6,7,10-12,46]. Thus, additional work is needed to understand the strengths and weaknesses of different teaching approaches, course durations, methods for providing longitudinal learning, leadership development, and mentorship. Furthermore, validated assessments of residents’ needs for leadership training, and the impact of training interventions on their knowledge, skills, attitudes, and behaviors, are needed to help improve the methodological rigor and reliability of course evaluations [5,31,32,47-51].

## Conclusion

In summary, we implemented a multi-session clinical leadership course pilot for internal medicine residents at a large academic medical center. This course was designed and taught internally, and included a number of unique elements, including business school style case studies about everyday clinical medicine. Initial course evaluations demonstrated improvements in participants’ knowledge and skills about clinical leadership, attitudes towards future leadership opportunities and training, and anticipated leadership behaviors.

## Additional file

Additional file 1: Table S1. Overview of Syllabus for Pilot Leadership Course. **Table S2.** Course Strengths. **Table S3.** Course Weaknesses and Suggestions for Improvement.
