# Supplementary material for: Implementing a pilot leadership course for internal medicine residents: design considerations, participant impressions, and lessons learned
Source: BMC Med Educ. 2014 Nov 30;14:257. doi: 10.1186/s12909-014-0257-2 (PMC4261637; doi:10.1186/s12909-014-0257-2)
Supplement: Additional file 1: Table S1. — Overview of Syllabus for Pilot Leadership Course. Table S2. Course Strengths. Table S3. Course Weaknesses and Suggestions for Improvement. [file 12909_2014_257_MOESM1_ESM.docx]

**Additional file 1**

**Table S1 Overview of Syllabus for Pilot Leadership Course**

| **Session Title (length)** | **Session Components (length)** | **Description of Content** | **Facilitator(s)** | **Required Reading** |
| --- | --- | --- | --- | --- |
| **Session 1: Introduction to Leadership and Leadership Styles (3 hrs)** | Introduction to Course and Leadership in Health Care (50 min) | - Overview of course goals, and logistics. - Interactive lecture addressing the following topics:  1. Traditional definitions of Leadership & Management; 2. Definition of clinical leadership and published evidence of its impact on quality, costs, and patient/provider satisfaction. | 1) Jessica Zeidman, MD (Internal Medicine Chief Resident)  2) Daniel M. Blumenthal, MD, MBA (3^rd^ Yr Internal Medicine Resident) | - Course and Syllabus - Bohmer, R. “Leading Clinicians and Clinicians Leading.” NEJM. 2013: 368(16): 1468-70. |
|  | **BREAK** (10 min) | | | |
|  | Large Group Case Discussion about “Sarah Schwartz” case (80 min) | - Discussion focused on following topics:   1) Identification of different leadership styles;  2) Strengths and weaknesses of these leadership styles;  3) Impact of stress on your leadership style;  4) Learning to adapt leadership styles to different situations. | 1) Kerri Palamara McGrath, MD (Primary Care Physician & Associate Director of MGH DOM Residency) | - Case: “Sarah Schwartz in the Massachusetts General Hospital’s Medical Intensive Care Unit.” |
|  | Small Group Meetings (40 min) | - “1^st^ leadership success” exercise: Participants   Reflect on 1^st^ successful leadership experience;  Present their experiences to their groups;  Identify common themes/challenges of these experiences. | A different group member facilitated each small group meeting. | None |
| **Session 2: Leadership Styles and Authentic Leadership**  **(2 hrs)** | Small Group Meetings (120 min) | - “Balancing authentic leadership styles with situational demands” exercise: Working individually, participants   1) Identified supervising residents’ major clinical leadership roles, and factors that may impact their leadership effectiveness in each role;  2) Described how they’d like colleagues to experience them as leaders; and formulated their authentic leadership styles.  They then discussed these topics as a group.   - “Leadership roles” exercise: In small groups, participants defined responsibilities of each of role identified above, and leadership styles likely to be more/less effective in each role. - Discussion of Johari’s Window exercise. - Discussion of leadership flexibility and situational awareness. | A different group member facilitated each small group meeting. | - “Johari’s Window” exercise |
| **Session 3: Leading with Emotional Intelligence (3 hrs)** | Large Group Discussion (80 min) | - Participants viewed scenes from the television show “ER” and discussed components of emotional intelligence exhibited in each scene. | 1) Ted Sterns, MD (Psychiatry Professor)  2) Justin Johnson, MD (Psychiatry Chief Resident) | - Goleman D. “What makes a leader?” Harvard Business Review. 1998: 92–102. |
|  | **BREAK** (10 min) | | | |
|  | Small Group Meetings (40 min) | - Role playing activity | Drs. Sterns and Johnson | None |
|  | Large Group Discussion (50 min) | - Individual reflection and large group discussion about role playing activity. | Drs. Sterns and Johnson | None |
| **Session 4: Leading Clinical Teams (3 hrs)** | Large Group Case Discussion about “A Day on the Bigelow” case (80 min) | - Discussion focused on identification of common challenges of leading a clinical team and strategies for addressing these challenges. | 1) Katrina Armstrong, MD (Oncologist & Chief of MGH DOM)  2) Daniel Hunt, MD (Clinician-educator & Chief, Hospital Medicine Unit) | - Case: “A Day on the Bigelow” |
|  | **BREAK** (10 min) | | | |
|  | Large Group Case Discussion about “A Day on the ID ISR” case (60 min) | - Discussion focused on following topics:   1) Managing relationships with superiors and non-MD clinicians; and  2) Approaches for addressing interdisciplinary conflict. | 1) Alberto Puig, MD, PhD (Clinician-educator & Associate Professor of Medicine) | - Case “A Day on the Infectious Disease (ID) Inpatient Subspecialty Rotation (ISR)” |
|  | Small Group Meetings (30 min) | - Participants completed post-course evaluations. | None | None |

**Table S2: Course Strengths**

| **General Topic** | **Specific Strength** | | **Number of Comments** | **Specific Examples** |
| --- | --- | --- | --- | --- |
| **Preparatory Work** | Case studies | | 3 | - “Well thought out written cases for large group discussions.” - “Focused but very high yield supplemental readings about leadership styles, emotional intelligence, leadership and management tips.” |
|  | Supplemental Reading | | 2 |  |
| **Large Group Meetings** | Moderators | | 5 | - “Some specific strengths were prepared facilitators who were well respected physicians who are themselves considered leaders.” - “Case discussions were terrific.” |
|  | Content of Discussions | | 5 |  |
| **Small Group Meetings** | Psychologically Safe Environment | | 4 | - “Provided an arena to discuss with peers concerns and hesitations about leadership” - “Small group interactions that helped allow for peer-based learning.” |
|  | Agendas and exercises | | 3 |  |
|  | Extended Evening Small Group Session | | 1 |  |
| **Course Timing** | | | 1 | - “Timing is perfect, right before we embark on junior year.” |
| **Course Facilitates Development of Practical Skills** | | | 4 | - “Practically addressed and anticipated issues we will face as junior residents.” |
| **Course Provides protected time and safe forum for reflection and discussion** | | | 4 | - “The small group sessions allowed very honest discussions with our classmates about the fears and leadership challenges we anticipate throughout the course.” |
| **The course overall (i.e. just having it)** | | | 2 | - “Having this course in the first place is a great strength.” |
| **Specific Course Content** | | Leadership Styles | 2 | - “A focus on leadership qualities/characteristics/styles with recognition of the differences.” - “Facilitated my understanding of my authentic leadership style and enabled me to reflect on situations where I am forced out of my authentic style and into other styles.” |
|  |  | Authentic Leadership | 1 |  |
|  |  | Emotional Intelligence | 1 |  |

**Table S3: Course Weaknesses and Suggestions for Improvement**

| **General Topic** | **Specific Weakness** | **Number of Comments** | **Examples of Feedback** |
| --- | --- | --- | --- |
| **Course Materials** | Syllabus unclear | 1 | - “My group floundered for quite some time trying to figure out exactly what we were supposed to do.” |
|  | Instructions for one or more exercises unclear | 2 |  |
| **Course workload** | Amount of preparatory work is at upper limit of what is reasonable (w/o causing fatigue) | 1 | - “I would not add more cases or reading or I think you would cause case fatigue during ACR.” |
| **Large Group Meetings** | Content and/or Structure of “Leading with Emotional Intelligence” Session (i.e. role playing, use of television show clips). | 7 | - “The emotional intelligence session provided a great platform to discuss the topic, but the lecture, videos, and role playing was quite redundant.” - “Coordinate more among speakers, so they know what topics have been covered to avoid unnecessary redundancy.” |
|  | Content and/or structure of “Leading Clinical Teams” Session | 2 |  |
|  | Redundancy in content addressed by different facilitators | 2 |  |
| **Small Group Meetings** | Conversations often strayed off topic | 3 | - “Conversations easily fell off topic. Exercises were not as helpful in focusing discussion as needed.” - “I feel that the small group discussions were not as helpful as the larger case discussions.” |
|  | Ineffective achieving objectives | 5 |  |
| **Course Organization and Logistics** | Remove Evening Session | 2 | - “Do not have an evening meeting.” - “I didn't like that it took away from ACR [outpatient] lectures.” |
|  | Course takes away from other learning opportunities | 1 |  |
| **Course Learning Methods** | Course should be multidisciplinary | 1 | - “One way in which this the course could move forward is by incorporating fellows and/or residents from other departments, as well as members of nursing staff, to be involved in the leadership discussion to get in-person feedback on what they view as effective leadership depending on the context and situation.” - “It would be nice if we could get some leadership/personality inventories so that we could learn more about our own leadership styles.” |
|  | Incorporate activities (e.g. simulation, role plays) to practice skills learned in course | 2 |  |
|  | Incorporate use of available personality inventories to facilitate self-awareness | 2 |  |
| **Continuing Education** | Course should be expanded beyond one month | 1 | - “Expand beyond one month.” |
